# Corrigendum to: real-time and noninvasive tracking of injectable hydrogel degradation using functionalized AIE nanoparticles

**DOI:** 10.1515/nanoph-2025-0306

**Published:** 2025-07-21

**Authors:** Mengdi Zhang, Zengliang Wang, Pengzhou Huang, Guanwei Jiang, Changpeng Xu, Wentao Zhang, Rui Guo, Wenqiang Li, Xintao Zhang

**Affiliations:** Department of Sports Medicine and Rehabilitation, National & Local Joint Engineering Research Center of Orthopaedic Biomaterials, Peking University Shenzhen Hospital, Shenzhen 518036, China; Shantou University, Shantou 515063, China; Department of Sports Medicine and Arthroscopy Surgery, Tianjin Hospital, No. 1 Wards Medicine, Tianjin 300210, China; Department of Sports Medicine and Rehabilitation, Peking University Shenzhen Hospital, Shenzhen 518036, China; Department of Orthopaedics, Guangdong Second Provincial General Hospital, Guangzhou 510317, China; Department of Biomedical Engineering, 47885 Key Laboratory of Biomaterials of Guangdong Higher Education Institutes, Guangdong Provincial Engineering and Technological Research Center for Drug Carrier Development, Jinan University, Guangzhou 510632, China; Guangzhou Sport University, Guangzhou 510500, China

The authors regret that the original version of our paper [1], unfortunately, contained incorrect pictures in Figure 7. It appeared that the photoacoustic images of the “2 % CCNC degraded in PBS at 336 h” group and the “2 % CCNC degraded in PBS/lysozyme at 524 h” group were derived from the same original source, even though they were intended to represent results from separate experiments. After consulting our original data, we confirmed that these errors occurred during the compilation of the figure.

**Figure 7:**
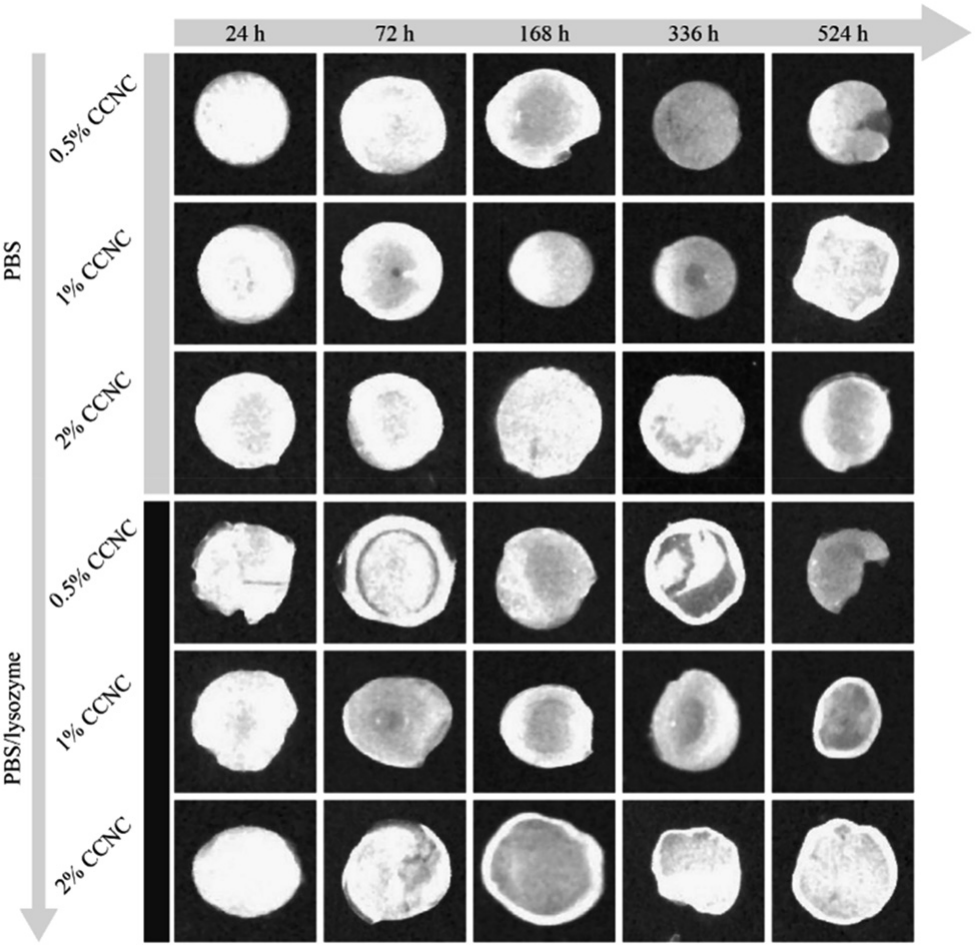
Representative photoacoustic imaging of *in vitro* degradation of DEX/CCNC/MPDA-TPE hydrogels with different content of CCNC in the absence of lysozyme or in the presence of lysozyme over time.

The correct version of Figure 7 was shown below.

These corrections are minor and do not alter the conclusions of the paper. The authors apologize for any inconvenience that the errors may have caused.
